# Klebsiella pneumoniae Liver Abscess as a Cause of Lung Entrapment: A Case Report and Literature Review

**DOI:** 10.7759/cureus.73264

**Published:** 2024-11-08

**Authors:** Martinha Vale, Raquel Azevedo, Inês Araújo, Catarina Araújo, Carla Maravilha, Ana Oliveira

**Affiliations:** 1 Internal Medicine, Unidade Local de Saúde de Braga, Braga, PRT; 2 Infectious Disease, Unidade Local de Saúde de Braga, Braga, PRT

**Keywords:** gallstones complication, klebsiella pneumoniae (kp), lung entrapment, pleural empyema, pyogenic liver abscess (pla)

## Abstract

A pyogenic liver abscess is a rare and potentially life-threatening disease, but the development of new treatment options has significantly improved its prognosis. Diseases resulting from *Klebsiella pneumoniae* infection are frequently associated with diabetes, malignancy, and hepatobiliary disease, particularly bile duct stones. This infectious agent is also frequently associated with severe complications, some of which are metastatic. We describe a complex case of a woman who presented with a pyogenic liver abscess due to *Klebsiella pneumoniae* infection, in which the atypical presentation delayed the diagnosis, and whose evolution was complicated by an uncommon and difficult-to-treat complication.

## Introduction

A pyogenic liver abscess is a rare and potentially life-threatening condition [1-4]. Risk factors include diabetes mellitus, hepatobiliary disease, pancreatic disease, colorectal disease, and neoplastic diseases such as colorectal cancer [1,4,5]. Abscess microbiology has also been shown to impact outcomes [1,4]. In recent years, the prevalence of pyogenic liver disease due to *Klebsiella (K.) pneumoniae*, a significant pathogen, has been increasing worldwide [4]. Diseases resulting from *Klebsiella pneumoniae* are frequently associated with diabetes, malignancy, and hepatobiliary disease, particularly bile duct stones [1,3,5,6]. This agent is also associated with a higher incidence of metastatic complications but with lower mortality as compared to other infectious organisms [1,6]. Treatments combining antibiotics with either percutaneous drainage or surgery have demonstrated reasonable cure rates [4,7].

Herein, we present a complex clinical case of pyogenic liver abscess that presented with chest pain radiating to the cervical region and right arm. Despite antibiotic treatment, the patient’s condition was still complicated by the formation of a pleural fistula and lung entrapment. We also review the clinical characteristics, etiology, microbiological isolates, and outcomes of pyogenic liver abscesses.

## Case presentation

A Brazilian woman in her fifties, diagnosed with symptomatic cholelithiasis and awaiting cholecystectomy, presented to the emergency department with central and oppressive chest pain radiating to the right cervical region and right arm. In the prior month, she also sought evaluation in the same emergency department due to discomfort in the right hypochondrium and fever. At that time, she underwent an abdominal ultrasound (US) that confirmed her known cholelithiasis without signs of acute cholecystitis. On the same day, she was discharged home and medicated with acetaminophen every eight hours for two days. Her symptoms resolved until she presented to the emergency room again, as described below.

The patient exhibited discomfort, sudoresis without fever (axillary temperature of 36.8ºC), tachycardia (142 beats per minute), tachypnea (32 cycles per minute), and hypertension (179/86 mmHg). On abdominal examination, she had pain in the right hypochondrium but without signs of peritoneal irritation. Her electrocardiogram revealed sinus tachycardia without ST alterations. Her blood workup showed leucocytosis (14.4x10*9/uL), thrombocytosis (619,000/uL), elevated C-reactive protein (150 mg/L; normal range < 5 mg/L), and elevated D-dimers (2295 ng/mL; normal range < 250 ng/mL). Acute coronary syndrome was excluded - troponin I 0.002 ng/mL (normal range < 0.045 ng/ml) and total creatine kinase 94 U/L (normal range 30-150 U/L).

**Figure 1:**
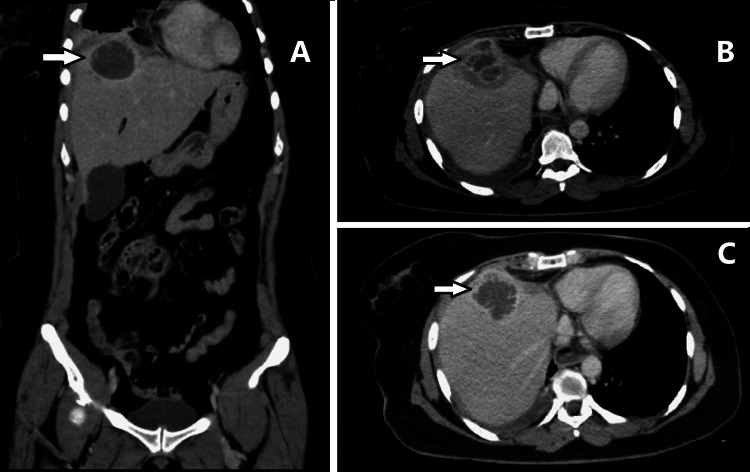
Thoracoabdominopelvic computed tomography scan showing a right hepatic abscess measuring 5 cm, with another contiguous component measuring 2 cm in the subphrenic region (white arrow)

To better distinguish the origin of the complaints, a thoracic, abdominal, and pelvic computed tomography (CT) was performed, which revealed a right hepatic/subphrenic abscess measuring approximately 5 cm, with a minor contiguous component measuring 2 cm subphrenic in segment VIII and a small right pleural effusion with atelectasis of the adjacent parenchyma (Figure 1); pulmonary embolism was excluded.

Blood cultures were collected, percutaneous drainage of the abscess was performed, and a US-guided abdominal drain was placed. The samples were sent for microbiological identification. Then, empirical antibiotic therapy was initiated with ceftriaxone (endovenous 2 g once daily) and metronidazole (endovenous 500 mg every eight hours). Her pain was treated with opioids. Despite treatment, her condition continued to worsen. On the third day of hospitalization, *Klebsiella pneumoniae* susceptible to piperacillin-tazobactam but resistant to third-generation cephalosporins was identified in the abscess drainage. As a result, her antibiotics were switched to a combination of piperacillin and tazobactam (4.5 g every six hours). The blood cultures were negative.

**Figure 2:**
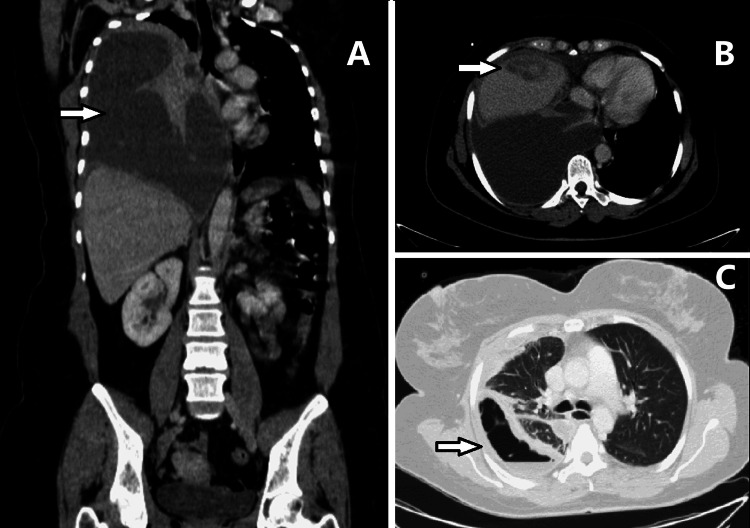
Computed tomography (CT) scan showing a pyogenic liver abscess complicated with pulmonary involvement (A) Coronal view of the thoracoabdominopelvic CT scan showing a massive right pleural effusion, with a partially loculated appearance and enhancement of the pleural leaflets, suggestive of empyema (arrow), causing a shift to the left side of the mediastinal structures; (B) Axial view of the thoracoabdominopelvic CT scan showing multiloculated empyema in the right pleural cavity. In the upper segments of the right hepatic lobe, there remained a heterogeneous area with a hypodense center concerning the known liver abscess (arrow) with a dimensional reduction, maintaining the apparent contiguity of the liver abscess with the adjacent pleural space; (C) Axial view of the thoracic CT scan showing right lung entrapment (arrow).

Despite the adjusted antibiotic therapy, the patient’s condition persisted to deteriorate, with hypoxic respiratory dysfunction and worsening of the inflammatory markers. The repeat CT scans exhibited a reduction in the dimensions of the abdominal abscess (29 x 55 mm) with the development of a significant complication - communication with the right lung pleural space with a large loculated pleural effusion compatible with empyema, forcing a mediastinal shift to the left and atelectasis of the ipsilateral lung (Figures 2A, 2B). A chest tube was placed, and a sample of the liquid was collected, confirming the diagnosis of empyema with negative cultures. The antibiotic regimen was maintained, and the patient’s condition began improving. Nevertheless, there was no full lung re-expansion, and the pleura stayed markedly thickened (Figure 2C).

**Figure 3:**
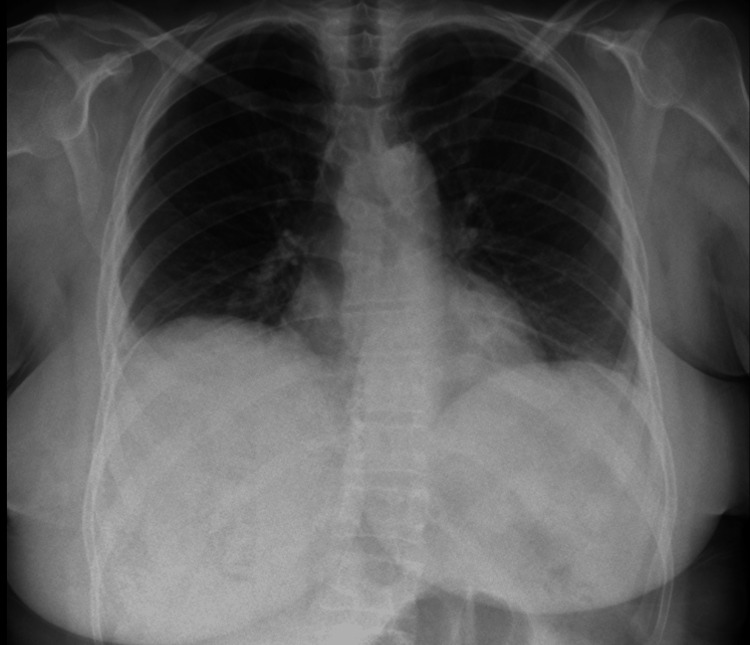
Chest X-ray with the resolution of the lung infectious process

Given the presence of pleural thickening with an incarcerated lung, the patient underwent pleural debridement and decortication. After the procedure, she completed a total of 30 days of antibiotic therapy with quick clinical and laboratory recovery and was discharged home from the hospital (Figure 3).

## Discussion

Pyogenic liver abscess is a rare disease, partially because the Kupffer cells in the liver confer some resistance to infections [3]. Nevertheless, the dual hepatic vasculature of the organ also makes it particularly vulnerable to forming abscesses [3]. The most common pathophysiological mechanism for liver abscess formation is the spread of infection via the hepatic portal vein [4,5,7]. Other mechanisms can also be involved, such as bacterial dissemination via the hepatic artery, continuity from the peritoneal cavity, superinfection of necrotic tissue, or ascending cholangitis [3-5]. *Klebsiella pneumoniae*, a common colonizer of the gastrointestinal tract, is frequently identified as a primary agent in biliary tract infections and is notably associated with severe complications. Although identifying the etiology of the condition is important, in about half of such cases, an etiology cannot be found, and they are known as cryptogenic abscesses [3-5,7].

The incidence of pyogenic liver abscesses is increased among adults, with a mean age of presentation of 57 years [4,7]. This increased incidence has been linked to a weakened immune system and increased comorbidities [4,5]. Diabetes mellitus is the most documented disease associated with pyogenic liver abscesses [4,5]. In our case, the patient presented with the condition at an age compatible with these statements. However, she did not present significant comorbidities such as diabetes.

Biliary tract disease is found in a significant percentage of pyogenic liver abscess cases, as was the case in our patient [3-5,7]. Additionally, in this particular case, cholelithiasis involvement was further confirmed by microbiological identification of *Klebsiella pneumoniae* in the products collected from the liver abscess, as this agent is frequently associated with the suspected etiology [6,8].

Typical manifestations of the disease include fever, right upper abdominal quadrant pain, nausea, and vomiting [2,4,6,8]. However, patients can also present with many other symptoms that can confound diagnosis such as pleuritic pain, cough, chest pain, palpitations, or weight loss [2,4-6,8]. This case exemplifies the entirely variable complaints and physical findings contributing to delayed diagnosis in such patients. The most predominant changes cited in laboratory investigations are elevated white blood cell count and significant elevation in C-reactive protein [4,6].

Radiological examination is essential in the diagnosis of pyogenic liver abscess [4]. Many patients present with changes in chest radiographs, such as right pleural effusion and elevated right hemidiaphragm, resulting in delayed diagnosis [4,5,7]. The right hepatic lobe is most often affected due to its dominant vascularization [3,4]. US and CT represent the gold standard of testing owing to their accuracy in identifying liver abscesses [4,5], with an ultrasound recommended as the initial diagnostic investigation [7]. However, US exams can have limitations, mainly when abscesses are small, isoechoic, and solitary [4]. This was possibly the case with our patient during her first visit to the emergency department, as the abdominal US found no abscess. 

No pathognomonic characteristics permit the distinction between agents based solely on imaging findings [6]. Nevertheless, some articles state that abscesses related to *Klebsiella pneumoniae* are generally single, thin-walled, multiseptated, solid masses with necrotic centers on CT [2,6].

Concerning treatment of pyogenic liver abscesses, a consensus has yet to be established [4]. If the patient's condition is life-threatening, management should follow the sepsis guidelines with early initiation of antibiotics [5]. Therapies combining percutaneous drainage with antibiotics or surgery with antibiotics are the two most accepted strategies, as they demonstrate good response rates [4]. Generally, imaging-guided percutaneous drainage is the first-line treatment in most units, whereas surgery is a second-line treatment owing to the effectiveness and lower invasiveness of the first option [2,4,6,8].

Antibiotics ideally should be initiated after product collection for microbiological studies, as long as the patient remains stable. First-line antibiotic options include monotherapy with extended-spectrum penicillin (e.g. piperacillin-tazobactam) or a combination of a third-generation cephalosporin (e.g. ceftriaxone) and metronidazole [6]. Although one of the most accepted antibiotic regimes, ceftriaxone plus metronidazole, was initiated in our case, this option was not optimal as the isolated agent presented a resistance profile to the cephalosporins. This incorrect choice of antibiotic highlights the importance of the microbiological collections, as they made it possible to adjust the antibiotic regimen. In addition, there is no consensus on the duration of antibiotic treatment, as it depends on the patient’s evolution. However, C-reactive protein could be considered the ideal marker for assessing treatment efficacy [4]. In our case, the duration of antibiotic therapy was based on the clinical, analytical, and imaging evolution of the patient and was particularly difficult to adjust given the complications that developed.

*Klebsiella pneumoniae *is a significant pathogen that is frequently associated with metastatic complications concomitant with liver abscess [1,2,8]. The most common complication is pleural effusion [3]. However, diseases associated with *Klebsiella pneumoniae* exhibit better outcomes than those of other bacteria, despite the higher incidence of complications [1,2]. Our patient developed a significant complication related to her pyogenic liver abscess, reinforcing the association between *Klebsiella pneumoniae* and complicated diseases. Fortunately, she recovered completely despite the complications. 

With the advent of percutaneous drainage and broad-spectrum antibiotics, the evolution of treatment options for pyogenic liver disease has led to significantly improved prognoses [2,7]. Mortality due to this disease is now rare except for patients with malignant disease [7].

## Conclusions

Although a pyogenic liver abscess is a life-threatening disease, recent advances in treatment options, including the possibility of percutaneous drainage and broad-spectrum antibiotics, have reduced the need for invasive techniques in treatment and improved prognosis. Despite pleural effusion being a common manifestation, this case is notable for its severity, with the progression to lung entrapment and the need for decortication. Finally, the case also emphasizes the usefulness of taking microbiological samples, allowing for the adjustment of antibiotic therapy, as the recommended empirical choices are not always the most appropriate treatment options.
